# Artificial Intelligence Algorithms for Expert Identification in Medical Domains: A Scoping Review

**DOI:** 10.3390/ejihpe14050078

**Published:** 2024-04-28

**Authors:** Sahar Borna, Barbara A. Barry, Svetlana Makarova, Yogesh Parte, Clifton R. Haider, Ajai Sehgal, Bradley C. Leibovich, Antonio Jorge Forte

**Affiliations:** 1 Division of Plastic Surgery, Mayo Clinic, Jacksonville, FL 32224, USA; 2 Robert D. and Patricia E. Kern Center for the Science of Health Care Delivery, Mayo Clinic, Rochester, MN 55905, USA; 3 Center for Digital Health, Mayo Clinic, Rochester, MN 55905, USA; 4 Department of Physiology and Biomedical Engineering, Mayo Clinic, Rochester, MN 55905, USA; 5 Department of Urology, Mayo Clinic, Rochester, MN 55905, USA

**Keywords:** expert finding, expert identification, knowledge management, artificial intelligence, machine learning, language model

## Abstract

With abundant information and interconnectedness among people, identifying knowledgeable individuals in specific domains has become crucial for organizations. Artificial intelligence (AI) algorithms have been employed to evaluate the knowledge and locate experts in specific areas, alleviating the manual burden of expert profiling and identification. However, there is a limited body of research exploring the application of AI algorithms for expert finding in the medical and biomedical fields. This study aims to conduct a scoping review of existing literature on utilizing AI algorithms for expert identification in medical domains. We systematically searched five platforms using a customized search string, and 21 studies were identified through other sources. The search spanned studies up to 2023, and study eligibility and selection adhered to the PRISMA 2020 statement. A total of 571 studies were assessed from the search. Out of these, we included six studies conducted between 2014 and 2020 that met our review criteria. Four studies used a machine learning algorithm as their model, while two utilized natural language processing. One study combined both approaches. All six studies demonstrated significant success in expert retrieval compared to baseline algorithms, as measured by various scoring metrics. AI enhances expert finding accuracy and effectiveness. However, more work is needed in intelligent medical expert retrieval.

## 1. Introduction

### 1.1. Background

The extensive amount of information available worldwide makes finding experts already knowledgeable in a specific field more desirable [1].

Expert finding is the task of locating highly knowledgeable individuals based on topic-specific expertise [2,3]. This task is valuable not only in academia but also in enterprise environments. Companies use expert-finding systems to save time and resources by quickly locating experts when specialized knowledge is needed, such as in ongoing projects or urgent situations [4].

Finding experts with substantial influence, authority, and expertise can be managed with different approaches, for example, by analyzing extensive unstructured data, heterogeneous network structures, or Linked Open Data movement (LOD) [5,6,7,8,9], based on temporal and topical expert profiles [10], in academic and social networks [11,12], or in trying to find a reviewer for peer reviews and funding [7,13,14,15,16]. 

Bots can help beginners and casual users find topic experts on different platforms by ranking users based on their expertise using word frequency and word embedding techniques. These bots are part of a broader trend of using artificial intelligence (AI), such as natural language processing (NLP) and TF-IDF algorithms, in expert-finding tools [17,18]. Such systems include chatbots that recommend experts based on their implementation and usage expertise. Additionally, methods like cosine similarity, key-concept collection, and the C-value method [19] are used to enhance expert retrieval effectiveness. Some researchers also consider the number of publications and experience in their expert identification approaches [20].

In medicine, as specialists face the medical information explosion [21] and the complexity of care continues to increase, efforts to provide personalized medical care require a substantial focus on effective referral systems. Referral rates in the United States more than doubled from 1999 to 2009; roughly 33% of patients receive a specialist referral yearly [22,23,24]. Furthermore, it has been established that physicians with more significant expertise in specific care processes decrease mortality and improve patient outcomes [25,26,27,28].

Electronic health records (EHRs) offer a wealth of valuable data, enabling context-adaptive algorithms to identify the most suitable experts for a patient’s specific healthcare needs [29]. However, manually identifying the most knowledgeable expert for a particular topic, especially in the context of disorders with limited data, is challenging. Traditional, non-AI algorithms may fall short of fully capturing the nuances of an individual’s expertise [30,31].

**Figure 1:**
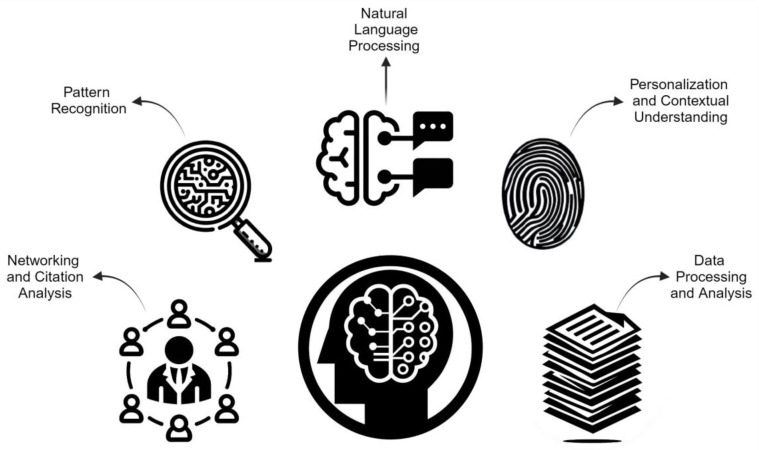
Advanced capabilities of AI compared to conventional algorithms. AI rapidly processes diverse data, including medical records and research, enabling quick expert identification through their contributions and impact. Advanced NLP in AI interprets complex medical language, and AI’s pattern recognition identifies emerging experts by analyzing data patterns and citation networks.

Through its capacity for data extraction and disease prediction, AI has exhibited promising outcomes in the field of personalized decision-making for patients [32,33,34,35]. AI can play a crucial role here, utilizing advanced clustering techniques to categorize experts based on similarities and semantic associations [36,37]—Figure 1. 

Although AI has been widely adopted by organizations to identify experts in various domains [38,39], the number of studies exploring the use of AI for expert identification in the medical and biomedical fields is limited. In this scoping review, we aim to explore various aspects of expert-finding models comprehensively. We will analyze the application of multiple AI algorithms and discuss their efficacy in medical expert retrieval. Additionally, we will assess the limitations of these models and offer recommendations for future research studies.

### 1.2. Research Questions

1. How does AI contribute to expert finding in various medical fields?
2. Can AI enhance the accuracy of expert identification?
3. How beneficial are AI models in medical expert finding?
4. What are the limitations associated with current approaches for finding experts using AI in medical fields?

## 2. Method

### 2.1. Search Strategy

In this study, we performed a comprehensive scoping review by systematically searching four databases: IEEE, PubMed, Web of Science, and Scopus. We included a search from the Google Scholar search engine to capture a broader range of literature that might not be indexed in traditional databases. Our search string ((“Artificial Intelligence”) OR (“Machine Learning”) OR (“Natural Language Processing”)) AND ((“expert finding”) OR (“expert retrieval”) OR (“expert identification”) OR (“expert recommendation”) OR (“expert selection”) OR (“expert discovery”) OR (“expert location”) OR (“expert search”)) was tailored to each specific database.

The search was conducted on 1st July 2023. For IEEE, PubMed, and Google Scholar, we limited our search to studies published between 2010 and 2023 to capture the most relevant and recent findings. For other databases, no time restrictions were applied due to the low number of search results. In the case of PubMed and Google Scholar, which yielded over 1000 results each, we reviewed the first 100 papers sorted by relevance. Additional studies were identified through a manual search of supplementary resources.

### 2.2. Study Eligibility and Selection Process

We identified relevant studies that specifically investigated the use of any type of artificial intelligence to identify or select experts within different medical domains without geographical restriction. Only empirical studies that provided data-driven insights and results were included, specifically original research articles presenting novel findings. We excluded studies that did not meet our inclusion criteria, articles in languages other than English, articles without full-text access, gray literature, and non-empirical studies (e.g., opinion pieces, commentaries, reviews, conference abstracts, or posters).

To ensure rigorous analysis, two independent researchers followed the Preferred Reporting Items for Systematic Reviews and Meta-Analyses (PRISMA) 2020 statement as the basis of our organization [40].

Figure 2.

After searching, papers from each mentioned database were selected using the search string. These papers were then reviewed chronologically, covering sections such as title, abstract, keywords, introduction, background, methodologies, findings, discussion, and conclusion to ensure a comprehensive evaluation. Articles were retrieved if the search phrase or a substring matched any components within them.

Subsequently, duplicate articles obtained from different databases were eliminated, and the collected papers were filtered using Endnote software (Version 21). The included reports were analyzed, and the results are presented descriptively, including numerical summaries and text.

## 3. Results

The primary search identified 571 relevant papers. Six studies were selected for inclusion in the review through screening and eligibility assessment. 

The studies were conducted between 2014 and 2020. Three studies reported the number of cases they examined [29,31,41], and all relied on textual sources as their primary data channel. One study [30] employed a generative probabilistic model to identify biomedical experts, while another used hybrid methods to construct a semantic term–expert linkage [37]. Among the studies, only two explicitly mentioned their labeling method, with automatic labeling used in one study [41] and manual labeling in another [29]. Please refer to Table 1 for a more comprehensive overview.

## 4. Discussion

### 4.1. Expert Finding and Artificial Intelligence: Why Do We Need AI?

In the vast landscape of resources, numerous experts try to share their knowledge with colleagues, organizations, and individuals seeking their insights on specific matters. The significant demand for information exchange among specialized individuals highlights the necessity for efficient tools to extract and identify these experts [3,42]. Expert profiles may be constructed by aggregating data from various web sources, such as publications, co-authors, citations, email communication, and LinkedIn information. This collected information can be further condensed into key descriptors that characterize the expert’s areas of expertise [42,43]. Expert-finding tools are utilized in various contexts, from major tech firms to medical institutions. However, these tools depend upon users to accurately assess their expertise in correlation to a predefined set of keywords. Moreover, the instruments must be regularly updated to correctly reflect any newly added skills by the experts [44].

Several requirements must be met to adopt systems as rapid, affordable, and confidential tools for expert identification. Firstly, experts should be identified through self-nominated specializations or by automatically extracting relevant information from various sources. Experts should be categorized according to the type and level of expertise, and the validity of their expertise should be confirmed by independent assessment of the depth and significance of their knowledge. Additionally, ranking these experts aids seekers in obtaining a more accurate understanding of their expertise level [45].

Due to the time-consuming and laborious task of manually creating expert databases, organizations have developed automated approaches to generate researcher profiles and identify potential experts through the use of AI techniques such as NLP and machine learning (ML) [46], which can be used to develop models for expert finding.

Multiple types of AI algorithms can be used in the expert finding domain [9,47,48,49,50,51,52,53], and several models have been developed to capture the relationship between query terms in expertise retrieval and expert candidates, including generative probabilistic models, graph-based models, voting models, and discriminative models [3].

### 4.2. Leveraging Artificial Intelligence for Efficient Expert Discovery

Document-based methods rank individuals based on their association with relevant documents rather than directly modeling their knowledge. They identify appropriate documents for a query and assign a ranking to candidates based on the document’s relevance score and the strength of the person’s association with it. This representation of a person consists of a weighted set of documents [3]. Various studies have employed different approaches for expert document collection, such as utilizing elementary units of experience [54]. Additionally, studies have applied various NLP and ML techniques, such as the BM2500 algorithm [55], statistical language models [44], term frequency–inverse document frequency (TF-IDF) [37,56], and LDA NLP models [37]. Multiple efforts are also underway to identify experts on communities of question-answering (CQA) websites using heterogeneous information such as question tags, content, and answer votes [6,51,52,53,57,58,59,60,61]. Zheng et al. and Rostami et al. showed that a deep learning framework could be used to find the most suitable experts in the question-answering community. In [57], the authors utilized DeepWalk, which takes inspiration from language modeling techniques and applies them to graphs. This method can create continuous vector representations that capture the relationships between elements in the network. Word-embedding-based convolutional neural network (CNN) architecture was another model that helped them capture the semantic and syntactic relationships between words [38,57]. It can be challenging for beginners and casual users on platforms like Discord to find experts in particular topics because it requires deep knowledge of the open-source community. To help with this, a bot named ExpertFinder uses word frequency and word embedding methods to create a list of users ranked by their expertise. ExpertFinder is a comprehensive map connecting authors with a list of words and their corresponding frequencies [62]. A similar approach was employed by the authors of [17], who introduced their bot using TF-IDF for sentence classification. Cerezo et al. designed an expert recommender chatbot that relied on the Discord API, TF-IDF algorithms for sentence classification and key-concept collection, and an expert recommendation system based on implementation and usage expertise [17]. In one study, query extension, TF-IDF calculation, and cosine similarity were employed for expert-finding purposes. The researchers explored applying the C-value method in NLP to identify and prioritize essential terms or phrases within the UvT Expert Collection corpus. This approach aimed to enhance the effectiveness of expert retrieval by considering relevant terms and their importance in the dataset [19]. In 2015, Hoon et al. presented “Classify”, a tool designed to facilitate rapid learning by leveraging existing sources of an expert’s expertise. Their approach incorporated measures such as TF-IDF and feature positioning within texts, eliminating the requirement for manual labeling of training data [18]. Furthermore, a multi-faceted approach was utilized by Afzal et al., where the experts were discovered by the number of their publications, quality of publications, citations, and experience [20].

### 4.3. Innovations in Medical Expert Finding through AI: Strategies and Results

Considerable efforts have been devoted to delivering optimal treatment recommendations to patients. In pursuit of this goal, scientists have explored various strategies, including clustering patients with shared manifestations, to effectively connect them with the most appropriate experts [63].

Although a notable amount of research has been dedicated to expert finding through AI across various fields, only a few studies have specifically focused on the medical and biomedical domains, particularly on automatically identifying physicians who are experts in specific areas. Furthermore, limited attention has been given to the perspective of primary care physicians and medical specialists.

**Figure 3:**
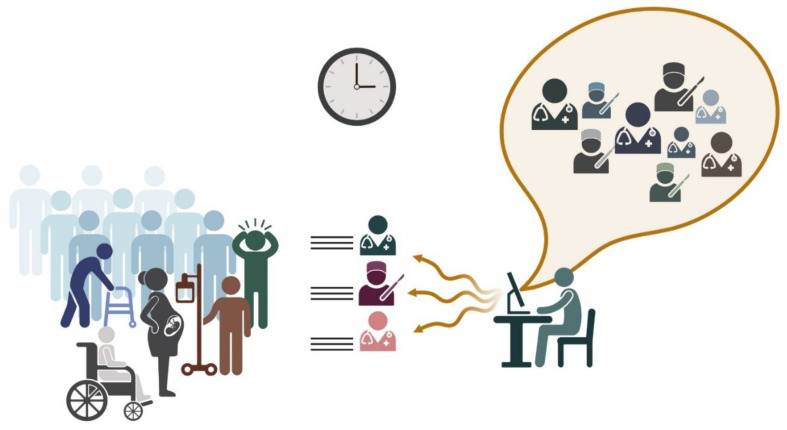
Utilizing artificial intelligence in the healthcare system to find the most expert specialist for patient referrals. The AI system streamlines the process by identifying and categorizing top specialists, ensuring patients are quickly matched with the most appropriate physician. This approach enhances efficiency, reduces wait times, and minimizes misallocations, leading to a more effective and less confusing healthcare experience.

In one study, Tekin et al. used an online learning model (Learn the EXpert (LEX)) as a context-adaptive tool to find the most suitable expert (human or clinical decision support systems (CDSS)) for patients. They defined the patient’s context as information related to their health condition and overall electronic medical records (demographic factors, drug history, etc.) and developed an algorithm to discover the most relevant contexts [29]. Organizing knowledge on patients or experts can help aggregate and link the information based on different criteria, such as the expert’s field of study or patients’ unique needs. This adaptation that uses AI for expert identification is essential in complex fields such as oncology, which is a significant area of medical research with multiple sub-specialties. Here, we can visualize expert relationships and evaluate the fusion similarity method’s performance in identifying experts in various topics, including breast cancer, endocrine gland cancer, liver cancer, and lung cancer [37]. However, Tekin et al.’s proposed algorithm provided comprehensive diagnostic accuracy measures for selected experts, assessing the optimal expert and decision-making. It also showed the importance of using an adaptable method in distributed medical settings, making it useful in non-centralized environments. This will be the most useful model if it dynamically updates expert accuracy estimates based on observed outcomes, ensuring a balanced workload distribution (Figure 3) [64].

However, final predictions still require a clinical examination by a physician. Their algorithm focused on diagnostic accuracy, potentially overlooking other aspects of a physician’s expertise [29]. Medical expert identification using knowledge organization systems (KOS) to make semantic connections between different aspects of information can be statistically beneficial, but it still overlooks the clinical aspects of patient care, assessing only academic contributions without capturing the essence of medical expertise, especially for complex patients [37]. This highlights the need to incorporate other factors, such as clinical experience and performance, to extract expertise in medicine more effectively. However, most of the efforts on expert identification by focusing on textual inputs and literature data show promising results. For example, in 2015, Wang et al. developed a novel BMExpert model to identify the biomedical experts from the MEDLINE database. They considered document relevance for the top 2000 papers and document–expert associations in their expert retrieval. They also used the documents’ importance as a factor for the first time. Unlike previous approaches, they did not give the same weight to all of the authors, and their model showed superior performance compared to JANE, eTBLAST, and GoPubMed in terms of mean average precision and precision metrics. While their model outperformed previous algorithms, comparing only three models, like the Tekin et al. study, decreases the chance for a more comprehensive evaluation. Also, using a specific dataset may limit the generalizability of the results to other domains [29,30]. 

**Figure 4:**
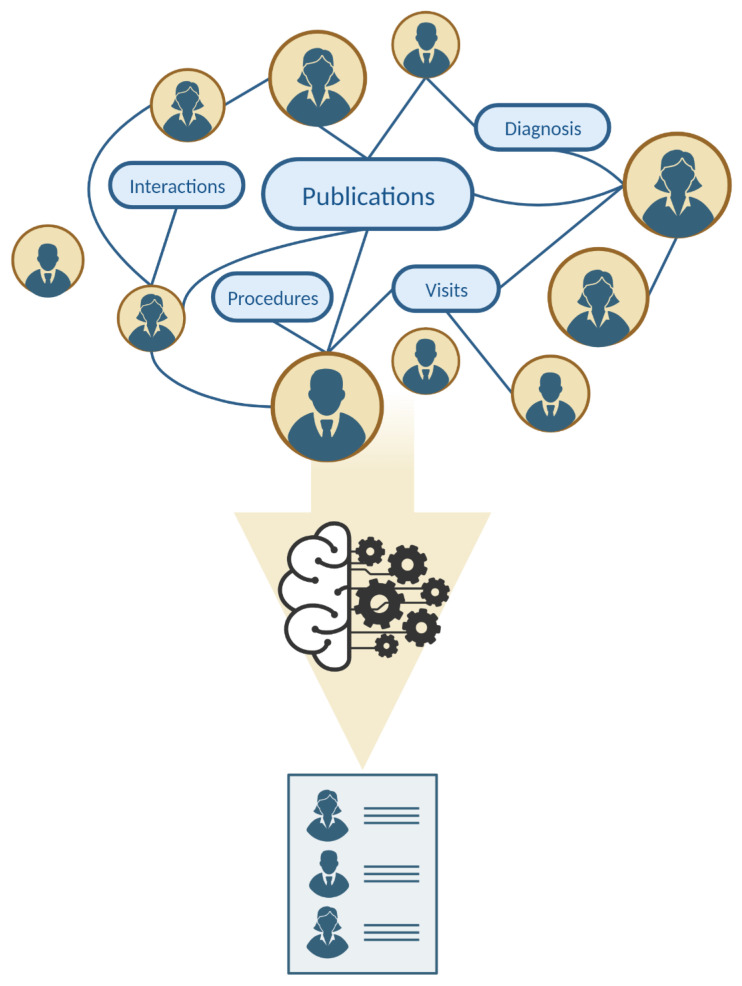
Medical expert finding using artificial intelligence.

Boeva et al. used the dynamic time warping (DTW) algorithm to match sequences, such as expert names and affiliation information. One year later, they applied three evolutionary clustering techniques to adapt the existing clustering solution to newly collected data elements. They created expert profiles by compiling the Medical Subject Headings (MeSH) terms associated with the PubMed articles of each author, measuring the semantic similarity for clustering experts into groups according to the degree of their expertise similarity. The two BCC algorithms (PivotBiCluster and Merge-Split PBC) consistently outperformed the partitioning-based algorithm, producing higher F-measure and SI scores on average [36,42]. In contrast, Bukowski et al. did not wholly rely on scientific publications. Still, they tried to assess the feasibility of profiling biomedical experts for interdisciplinary research and development (R&D) collaborations using publications and patents. Using an ML-based approach, the recommendation system achieved a high mean average precision of 89% compared to scientometric measures [41]. The same limitations apply to Wang et al.’s study, which utilized five ML methods to identify expert physicians in rare disorders by analyzing their publication history. They achieved a high classification accuracy in identifying physician experts from GeneReviews chapters and reputable peer-reviewed summaries of various inherited conditions. Additionally, their approach led to the prediction of 41,129 new associations between diseases and experts. Their obtained random forest model demonstrated the best performance among their ML methods and the baseline measure [31]. Using GeneReviews chapters as an external validation dataset strengthens their study’s findings, but reliance on a limited dataset introduces potential bias. Additionally, their focus on a specific set of rare diseases limits the applicability of their results to other disorders, particularly those with more extensive population involvement (Figure 4) [65]. 

There has been a significant shift towards more complex and versatile systems, beginning with basic linear models designed for straightforward, homogenous data problems [29]. This evolution is exemplified by BMExpert, which initially applied NLP techniques to address more intricate data structures [30]. Developing hybrid models that combine multiple machine learning methods, such as LogR, SVM, and others, has been critical in tackling complex data. These models have improved their accuracy and ability to adapt, particularly in processing unstructured data and pattern recognition [31]. Earlier models often required reprocessing the entire dataset for updates, a difficult task, especially with large datasets. However, recent advancements have led to algorithms that can update adaptively, saving substantial computational resources and time. A notable improvement in these models is seen in their clustering approach, where they can modify existing clustering solutions to include new data without reprocessing the entire dataset [36]. With the development of models capable of understanding the semantic meaning of data, there has been an improvement in contextually understanding phrases, leading to more accurate recommendations [36,37]. Furthermore, using Feedforward ANN algorithms in these advanced models enables them to handle various datasets, including publications, patents, and project descriptions. These deep learning-capable models can identify patterns and connections previously undetectable by earlier models [36,41].

### 4.4. Key Recommendations for Future Research

1. Future research should focus on designing algorithms that track changes in expert profiles and expertise to capture the most recent skills. In the context of the physician–patient relationship, incorporating the latest changes in patient profiles can facilitate accurate and timely referral actions.
2. It is recommended that the algorithms be evaluated across different medical fields and with a more significant number of expert-seekers to enhance generalizability.
3. The efficiency and accuracy of the models should be assessed in real-world patient scenarios to find the weaknesses of the algorithms.
4. Efforts should be made to develop more comprehensive models considering multiple aspects of an expert’s academic and clinical experience. This includes exploring the connections between different experts and their performed procedures to extract more precise information.
5. It is advisable to compare the proposed models with a broader range of existing models to obtain a more accurate estimation of their accuracy and performance.

### 4.5. Strengths and Limitations

This review is the first in the field of expert finding in the medical domain. We conducted a rigorous search and screened papers based on predefined criteria. Our evaluation focused on the methodologies and obtained models in the included studies. We provided directions for future studies in this field, aiming to enhance further the effectiveness and applicability of AI techniques in expert-finding tasks.

Our review did not explore privacy and information security challenges in medical expert finding. Our primary goal was to assess the applicability of different approaches rather than comparing essential algorithm performance. Due to the limited number of publications in this area, the generalizability of our findings may be limited compared to other aspects of expert finding. Additionally, we did not focus on doctor recommendation systems based on ratings, as they may not fully consider the expertise of physicians.

## 5. Conclusions

In this review, we analyzed various AI-based methodologies for expert finding in medical fields, addressing our primary research questions. The studies reviewed demonstrated the effectiveness of AI algorithms, yielding accuracy between 79.5% and 83.32% and ROC AUC scores from 0.71 to 0.88, compared to established benchmarks. These results underscore the potential of AI in enhancing the accuracy and efficiency of medical expert identification, which is crucial for informed decision-making and improving patient referral processes. However, it is vital to acknowledge the limitations inherent in these studies, such as the potential for biases in dataset selection and the challenges in generalizing findings across different medical domains. These studies’ varying methodologies and contexts also highlight the need for further research to refine AI algorithms and expand their applicability.

Future research should focus on developing more versatile and adaptive AI models that cater to the dynamic nature of medical expertise and patient needs. Exploring the integration of diverse data sources, including clinical performance metrics and patient outcomes, could offer a more holistic approach to expert finding. Integrating AI in medical expert finding holds significant promise for transforming healthcare delivery. By continuing to refine these technologies and understanding their implications, we can better harness AI’s capabilities to meet the evolving demands of patient care and medical research.

## Figures and Tables

**Figure 2:**
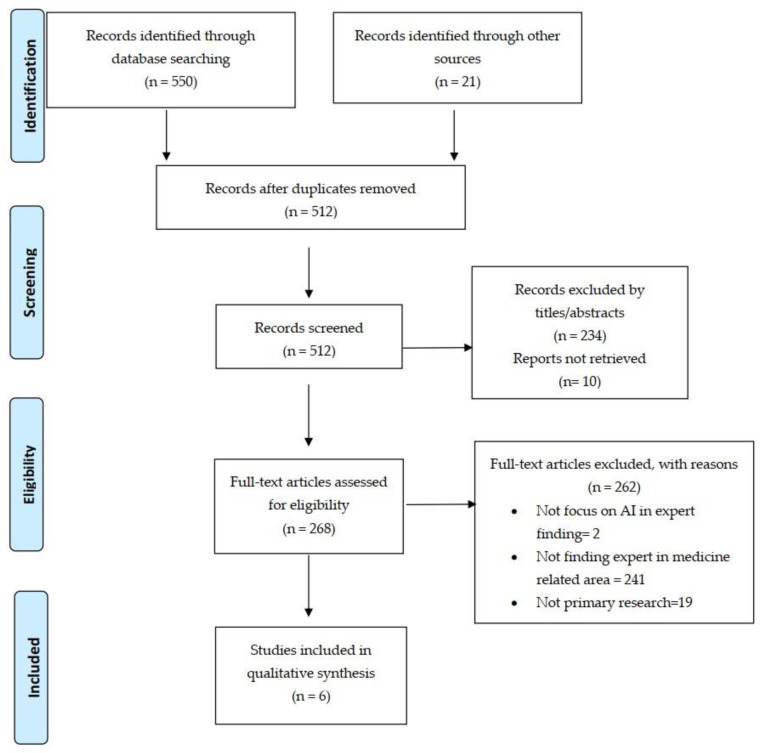
PRISMA flow diagram. Study selection process. The diagram outlines steps from identifying to including studies, showing record filtration, and reasons for exclusion.

**Table 1:** Key information of the included studies. Abbreviations: Acc (Accuracy); ANN (artificial neural networks); AP (average precision); DFG (German Research Foundation); DPMA (German Patent and Trademark Office); EPO (European Patent Office); LDA (Latent Dirichlet Allocation); LEX (Learn the Expert); LogR (logistic regression); MAP (mean average precision); ML (machine learning); MS PBC2 (Merge-Split PivotBiCluster); NB (Naive Bayes); NLP (natural language processing); NIH (National Institutes of Health); Nnet (neural network); P@50 (average precision at 50); PBC (PivotBiCluster); PB (partitioning-based); RAKE (Rapid Automatic Keyword Extraction); RF (random forest); SI (Silhouette Index); TF-IDF (Term Frequency–Inverse Document Frequency); WOS (Web of Science).

Author and Year	Number of Cases	Task	Channel	Inputs	Dataset	Technique: Algorithm	Kind of Model	Validation Method	Evaluation Method
Tekin et al., 2014 [29]	45,450 patients	Expert finding Diagnostic evaluation Context recognition	Textual sources	Context vectors Expert contexts Partition of expert context space Diagnostic actions	UCLA Radiology’s Breast Cancer Dataset	ML: LEX	Adaptive online learning algorithm	Experimental validation	N = 1000: Acc = 80.03% N = 3000: Acc = 82.49% N = 5439: Acc = 83.32%
Wang et al., 2015 [30]	N\A	Expert finding and ranking	Textual sources	Topic Query Author features Biomedical literature (MEDLINE)	ISMB conference program committee members (2012–2014)	ML: BMExpert	Language model	Experimental validation	P@50 = 6.71%, AP = 4.14%
Wang et al., 2017 [31]	209,110 Disease–author associations	Rare diseases expert finding	Textual sources	Known and unknown disease–expert associations Publication and author features	GeneReviews OMIM.org	ML: LogR, SVM, RF, NB, Nnet	Classifier Neural network	5-fold cross-validation	Baseline measure: ROC AUC = 0.69 RF: ROC AUC = 0.88, Acc = 79.50%, Precision = 80%, Recall = 78% LogR: ROC AUC = 0.86 NB: ROC AUC = 0.78 SVM: ROC AUC = 0.71 Nnet: ROC AUC = 0.87
Boeva et al., 2018 [36]	N\A	Expert finding and expertise retrieval	Textual sources	Set of experts Subject categories Set of recently extracted experts Biomedical literature	PUBMED	NLP: PB, PBC, MS PBC2	Clustering model	Experimental validation	Experiment 1: F-measure: PB: 0.618 PBC: 0.640 MS PBC2: 0.628 SI Score: PB: −0.145 PBC: −0.139 MS PBC2: −0.139 Experiment 2: F-measure: PB: 0.321 PBC: 0.308 MS PBC2: 0.302 SI Score: PB: 0.137 PBC: 0.164 MS PBC2: 0.159
Pei-yan et al., 2019 [37]	N\A	Semantic term–expert linkage construction	Hybrid expertise sources Social networks Textual sources	Expert and literature metadata, expert knowledge organization tools, semantic labels, RDF framework, TF-IDF, and LDA analysis results	Chinese library classification for oncology literature	NLP: TF-IDF, LDA	Knowledge-based system	Experimental validation	Recall ratio = 50% to 70.6% Accuracy ratio = 64.9% to 78.8% F value = 60% to 73.3%
Bukowski et al., 2020 [41]	Product-related model: 1398 cases Technological model: 2191 cases Clinical model: 3444 cases	Expert profiling and finding	Hybrid expertise sources Textual sources	Scientific publications Patents R&D project descriptions Online databases	PubMed WoS NIH DPMA EPO DFG	ML: SVM NLP: RAKE Feedforward ANN	Classifier Extracting method Neural network	Self-assessment External assessment 10-fold cross-validation	Selection: Overall F1-score 57% to 63%. F1-score weighted profiling without patent = 80% Ranking: Overall MAP 89% MAP based on scientometric measures = 41%

## Data Availability

Not applicable.
